# Supplementary material for: In vivo mapping of striatal neurodegeneration in Huntington’s disease with Soma and Neurite Density Imaging
Source: eLife. 2026 Aug 11;14:RP107661. doi: 10.7554/eLife.107661 (PMC13461150; doi:10.7554/eLife.107661)
Supplement: Supplementary file 2 [file elife-107661-supp1.docx]

| **Supplement Table 1a. Demographic and clinical information per HD-ISS stage** | | | | | | | | | | |
| --- | --- | --- | --- | --- | --- | --- | --- | --- | --- | --- |
|  | **HD-ISS 0** | | **HD-ISS 1** | | **HD-ISS 2** | | **HD-ISS 3** | | **HD-ISS NA** | |
| **Measure** | **N** | **Mean (SD)** | **N** | **Mean (SD)** | **N** | **Mean (SD)** | **N** | **Mean (SD)** | **N** | **Mean (SD)** |
| Age | 4 | 37.75 (16.30) | 9 | 47.11 (12.36) | 5 | 48.80 (10.83) | 12 | 49.17 (12.20) | 26 | 42.65 (14.97) |
| Sex, Female (%) | 4 | 3 (75%) | 9 | 5 (56%) | 5 | 1 (20%) | 12 | 3 (25%) | 26 | 13 (50%) |
| Education (years) | 2 | 15.50 (0.71) | 7 | 15.71 (1.38) | 5 | 14.60 (3.71) | 12 | 13.00 (2.17) | 12 | 14.00 (3.13) |
| MOCA | 4 | 25.50 (1.91) | 9 | 28.00 (1.87) | 5 | 26.00 (2.45) | 11 | 23.73 (3.20) | 26 | 27.35 (3.94) |
| TOPF | 4 | 60.25 (5.38) | 9 | 50.22 (14.03) | 5 | 44.20 (14.10) | 12 | 43.25 (10.34) | 26 | 51.92 (13.67) |
| UHDRS-TFC | 4 | 13.00 (0.00) | 9 | 12.78 (0.44) | 5 | 13.00 (0.00) | 12 | 10.92 (1.16) | 24 | 12.12 (1.30) |
| UHDRS-TMS | 4 | 1.50 (3.00) | 9 | 4.67 (4.90) | 5 | 10.20 (1.48) | 12 | 29.33 (20.38) | 18 | 6.22 (11.34) |
| CAG | 4 | 43.00 (2.16) | 9 | 41.89 (1.17) | 5 | 42.00 (1.00) | 12 | 43.67 (3.14) | 20 | 40.40 (2.56) |
| CAP100 | 4 | 72.34 (15.89) | 9 | 85.50 (16.98) | 5 | 91.09 (25.58) | 12 | 99.70 (16.68) | 20 | 65.84 (18.50) |
| SDMT | 2 | 69.00 (5.66) | 7 | 54.29 (13.03) | 5 | 45.60 (8.73) | 12 | 33.83 (12.65) | 12 | 48.00 (12.62) |
| **Abbreviations**: **CAG**: Cytosine Adenine Guanine; **CAP**: CAG-Age-Product; **HC**: Healthy controls; **HD**: Huntington’s disease; **HD-ISS**: HD-Integrated Staging System; **MOCA**: Montreal Cognitive Assessment; **SD**: Standard deviation; **SDMT**: Symbol Digit Modalities Task; **TFC**: Total Functional Capacity; **TMS**: Total Motor Score; **TOPF**: Test of Premorbid Functioning; **UHDRS**: United Huntington’s Disease Rating Scale | | | | | | | | | | |

| **Supplement Table 1b** Descriptive statistics for motor outcome measures | | |
| --- | --- | --- |
|  | **HD group** | |
| **Task**  *Outcome measure* | **N** | **Mean (SD)** |
| **Speeded tapping** |  |  |
| *Left finger; Area under the curve (N-s)* | 38 | 0.13 (0.20) |
| *Right finger; Area under the curve (N-s)* | 38 | 0.07 (0.09) |
| *Left finger; Inter-onset interval (s)* | 38 | 0.26 (0.12) |
| *Right finger; Inter-onset interval (s)* | 38 | 0.23 (0.08) |
| *Left foot; Inter-onset interval (s)* | 37 | 0.37 (0.20) |
| *Right foot; Inter-onset interval (s)* | 38 | 0.35 (0.18) |
| **Metronome tapping; Absolute deviation from pace (s)** |  |  |
| *Left finger; Paced; Fast* | 38 | 0.07 (0.06) |
| *Left finger; Paced; Slow* | 38 | 0.13 (0.14) |
| *Left finger; Unpaced; Fast* | 38 | 0.07 (0.07) |
| *Left finger; Unpaced; Slow* | 38 | 0.15 (0.15) |
| *Right finger; Paced; Fast* | 38 | 0.07 (0.06) |
| *Right finger; Paced; Slow* | 38 | 0.13 (0.12) |
| *Right finger; Unpaced; Fast* | 38 | 0.08 (0.06) |
| *Right finger; Unpaced; Slow* | 38 | 0.16 (0.17) |
| *Left foot; Paced; Fast* | 36 | 0.07 (0.05) |
| *Left foot; Paced; Slow* | 35 | 0.16 (0.13) |
| *Left foot; Unpaced; Fast* | 36 | 0.08 (0.09) |
| *Left foot; Unpaced; Slow* | 35 | 0.17 (0.14) |
| *Right foot; Paced; Fast* | 37 | 0.08 (0.04) |
| *Right foot; Paced; Slow* | 37 | 0.16 (0.12) |
| *Right foot; Unpaced; Fast* | 37 | 0.09 (0.09) |
| *Right foot; Unpaced; Slow* | 37 | 0.16 (0.13) |
| **Pointing (dominant hand)** |  |  |
| *Target frequency over trial (Hz)* | 38 | 2.62 (0.65) |
| **Pointing (dominant) and tapping (non-dominant)** |  |  |
| *Finger tapping area under the curve (coefficient of variation %)* | 38 | 0.76 (0.37) |
| *Finger tapping area under the curve (N-s)* | 38 | 0.31 (0.43) |
| *Finger tapping inter-onset interval (s)* | 38 | 0.39 (0.19) |
| *Frequency over trial (Hz)* | 38 | 3.08 (1.07) |
| *Pointing target frequency over trial (Hz)* | 38 | 2.62 (0.66) |
| **Abbreviations:** **Hz:** Hertz; **N:** Newton; **s:** Seconds |  |  |

| **Supplement Table 1c.** Rotated Component Loadings on the Q-motor Outcome Measures | |
| --- | --- |
| **Task**  *Outcome measure* | **Q-motor Factor** |
| **Speeded tapping** |  |
| *Left finger; Area under the curve (N-s)* | **0.707** |
| *Right finger; Area under the curve (N-s)* | **0.723** |
| *Left finger; Inter-onset interval (s)* | **0.772** |
| *Right finger; Inter-onset interval (s)* | **0.859** |
| *Left foot; Inter-onset interval (s)* | **0.798** |
| *Right foot; Inter-onset interval (s)* | **0.893** |
| **Metronome tapping; Absolute deviation from pace (s)** |  |
| *Left finger; Paced; Fast* | **0.875** |
| *Left finger; Paced; Slow* | **0.832** |
| *Left finger; Unpaced; Fast* | **0.936** |
| *Left finger; Unpaced; Slow* | **0.913** |
| *Right finger; Paced; Fast* | **0.730** |
| *Right finger; Paced; Slow* | **0.672** |
| *Right finger; Unpaced; Fast* | **0.886** |
| *Right finger; Unpaced; Slow* | **0.865** |
| *Left foot; Paced; Fast* | **0.823** |
| *Left foot; Paced; Slow* | **0.883** |
| *Left foot; Unpaced; Fast* | **0.884** |
| *Left foot; Unpaced; Slow* | **0.900** |
| *Right foot; Paced; Fast* | **0.818** |
| *Right foot; Paced; Slow* | **0.885** |
| *Right foot; Unpaced; Fast* | **0.890** |
| *Right foot; Unpaced; Slow* | **0.740** |
| **Pointing (dominant hand)** |  |
| *Target frequency over trial (Hz)* | **-0.525** |
| **Pointing (dominant) and tapping (non-dominant)** |  |
| *Finger tapping area under the curve (N-s)* | **0.734** |
| *Finger tapping inter-onset interval (s)* | **0.788** |
| *Frequency over trial (Hz)* | **-0.756** |
| *Pointing target frequency over trial (Hz)* | **-0.568** |
| All loadings were significant (>0.5 and < -0.5) and are highlighted in bold.  **Abbreviations:** **Hz:** Hertz; **N:** Newton; **s:** Seconds |  |

| **Supplement Table 1d. Descriptive statistics and non-parametric pairwise comparisons for SANDI indices (significant for total HD sample vs HC) between HD-ISS 0-1, HD-ISS 2-3, and Healthy Controls.** | | | | | | | |
| --- | --- | --- | --- | --- | --- | --- | --- |
|  | | **Descriptive statistics Mean (SD)** | | | **Pairwise comparisons**  **Statistic, (p-value)**  **Effect size [CI 95%]** | | |
| **Microstructural variable** | **Region of interest** | **HD-ISS 0-1**  **N=13** | **HD-ISS 2-3**  **N=17** | **HC**  **N=57** | **HD-ISS 0-1 vs HC** | **HD-ISS 2-3 vs HD-ISS 0-1** | **HD-ISS 2-3 vs HC** |
| Apparent soma density | Caudate | 0.45 (0.02) | 0.41 (0.07) | 0.47 (0.02) | 244 (0.057)  -0.228 [-0.43 – -0.02] | **56 (0.024)  -0.416 [-0.70 – -0.10]** | **119 (<0.001)  -0.546 [-0.69 – -0.38]** |
|  | Putamen | 0.42 (0.03) | 0.38 (0.05) | 0.43 (0.03) | 294 (0.251)  -0.138 [-0.35 – -0.01] | **53 (0.017)  -0.439 [-0.70 – -0.10]** | **174 (<0.001)  -0.464 [-0.64 – -0.25]** |
|  | Pallidum | 0.22 (0.03) | 0.19 (0.03) | 0.24 (0.04) | **229 (0.033)  -0.255 [-0.45 – -0.04]** | 64 (0.054)  -0.355 [-0.69 – -0.03] | **158 (<0.001)  -0.488 [-0.65 – -0.30]** |
| Apparent soma size | Caudate | 9.72 (0.17) | 9.88 (0.28) | 9.55 (0.13) | **587 (0.001)  0.391 [0.14 – 0.57]** | 150 (0.103)  0.302 [0.02 – 0.62] | **842 (<0.001)  0.534 [0.32 – 0.69]** |
|  | Putamen | 9.62 (0.19) | 9.77 (0.30) | 9.41 (0.14) | **597 (<0.001)  0.409 [0.19 – 0.60]** | 146 (0.143)  0.271 [0.02 – 0.60] | **832 (<0.001)  0.519 [0.27 – 0.68]** |
|  | Pallidum | 8.79 (0.52) | 8.11 (1.44) | 8.96 (0.51) | 263 (0.106)  -0.194 [-0.39 – -0.01] | **59 (0.033)  -0.394 [-0.69 – -0.06]** | **157 (<0.001)  -0.489 [-0.66 – -0.31]** |
| Extracellular signal fraction | Caudate | 0.37 (0.01) | 0.38 (0.03) | 0.36 (0.01) | **638 (<0.001)  0.483 [0.31 – 0.62]** | 148 (0.121)  0.287 [0.02 – 0.64] | **769 (<0.001)  0.425 [0.16 – 0.63]** |
|  | Putamen | 0.35 (0.01) | 0.37 (0.04) | 0.33 (0.01) | **597 (<0.001)  0.409 [0.22 – 0.56]** | 147 (0.132)  0.279 [0.02 – 0.62] | **776 (<0.001)  0.435 [0.17 – 0.64]** |
|  | Pallidum | 0.35 (0.04) | 0.38 (0.04) | 0.33 (0.02) | **517 (0.027)  0.264 [0.03 – 0.49]** | 157 (0.054)  0.355 [0.04 – 0.67] | **881 (<0.001)  0.592 [0.43 – 0.71]** |
| Extracellular diffusivity | Caudate | 1.39 (0.09) | 1.58 (0.24) | 1.42 (0.09) | 298 (0.277)  -0.131 [-0.36 – -0.01] | **169 (0.015)  0.447 [0.11 – 0.72]** | **697 (0.006)  0.317 [0.06 – 0.55]** |
|  | Putamen | 1.47 (0.12) | 1.63 (0.17) | 1.42 (0.09) | 490 (0.072)  0.216 [0.02 – 0.44] | **169 (0.015)  0.447 [0.13 – 0.71]** | **842 (<0.001)  0.534 [0.36 – 0.67]** |
|  | Pallidum | 1.67 (0.19) | 1.80 (0.18) | 1.53 (0.11) | **569 (0.003)  0.358 [0.09 – 0.56]** | 156 (0.060)  0.348 [0.04 – 0.65] | **893 (<0.001)  0.610 [0.47 – 0.72]** |
| Abbreviations: CI, Confidence Intervals.  Pairwise comparisons were conducted with Mann–Whitney U tests; effect sizes are rank–biserial correlations (signed) with 95% CIs.  Bold indicates p < 0.05. | | | | | | | |
