# Supplementary material for: In vivo mapping of striatal neurodegeneration in Huntington’s disease with Soma and Neurite Density Imaging
Source: eLife. 2026 Aug 11;14:RP107661. doi: 10.7554/eLife.107661 (PMC13461150; doi:10.7554/eLife.107661)
Supplement: Peer review history

# Editor report: eLife Assessment

**DOI:** 10.7554/eLife.107661.3.sa0

**Authors:** Jason P Lerch

**Affiliations:** University of Oxford United Kingdom

**Keywords:** Compelling, Fundamental

This **fundamental** manuscript presents a novel application of the SANDI (Soma and Neurite Density Imaging) model to study microstructural alterations in the basal ganglia of individuals with Huntington's disease (HD). The **compelling** methods are, to our understanding, the first application of SANDI to neurodegenerative diseases, provide strong evidence for HD-related neurodegeneration in the striatum, account significantly for striatal atrophy, and correlate with motor impairments. The integration of novel diffusion acquisition and modelling methods with multimodal behavioural data are both of high value in their own right, and create a framework for future studies.

# Referee report: Reviewer #1 (Public review)

**DOI:** 10.7554/eLife.107661.3.sa1

(1) In this study, the authors aimed at characterizing Huntington's Disease (HD) - related microstructural abnormalities in the basal ganglia and thalami as revealed using Soma and Neurite Density Imaging (SANDI) indices (apparent soma density, apparent soma size, extracellular water signal fraction, extracellular diffusivity, apparent neurite density, fractional anisotropy and mean diffusivity).

(2) The study implements a novel biophysical diffusion model that extends up-to-date methodologies and presents a significant potential for quantifying neurodegenerative processes of the grey matter of the human brain in vivo. The authors comment on the usefulness of this technique in other pathologies, but they exemplify only with multiple sclerosis. Further development of this, building evidence should be provided.

(3) Study found that HD-related neurodegeneration in the striatum accounted significantly for striatal atrophy and correlated with motor impairments. HD was associated with reduced soma density, increased apparent soma size and extracellular signal fraction in the basal ganglia, but not in the thalami. Additionally, these affects were larger at manifest stage.

(4) The results of this work demonstrate the impact of HD on basal ganglia and thalami which can be further explored as a non-invasive biomarker of disease progression. Additionally, the study shows that SANDI can be used to explore grey matter microstructure in a variety of neurological conditions.

Comments on revised version.

I have no further comments. Thank you

# Referee report: Reviewer #3 (Public review)

**DOI:** 10.7554/eLife.107661.3.sa2

Summary:

Ioakeimidis and colleagues studied miscrostructural abnormalities in N=56 Huntington's disease (HD) patients compared to N=57 normative controls. The authors used a powerful MRI Connectom scanner and applied the SANDI model to estimate the soma size, neurite size, soma density, and extracellular fraction in key subcortical nuclei related to HD. In the striatum, they found decreased soma density and increased soma size, which also seemed to become more pronounced in advanced HD individuals in the final exploratory analyses. The authors conducted important analyses to find whether the SANDI measures correlate with clinical scores (i.e., QMotor) and whether the variance of the striatal volume is explained by the SANDI measures. They found a relationship of SANDI measures to both.

Strengths:

The study is both innovative and of high interest for the HD community. The authors provide a rich pool of statistical analyses and results which anticipate the questions that may emerge in the HD research community. Statistics are carefully chosen and image processing is done with state-of-the-art methods and tools. The sample size gives sufficient credibility to the findings. Altogether, I think this study sets a milestone in the attempts of the HD community to understand neuropathological processes with non-invasive methods, and extends the current knowledge of microstructural anomalies identified in HD with diffusion MRI. More importantly, the newly identified anomalies in soma size and soma density open new avenues for studying these biological effects further, and perhaps develop these biomarkers for use in clinical trials.

Weaknesses:

(1) An important question is whether the SANDI measures, which require an expensive scanner and elaborate processing, are better biomarkers than the more traditional DTI measures. Can the authors compare the effect size of FA/MD with SANDI measures. In some of the plots and tables, FA/MD seem to have comparable, if not higher, correlations with QMotor or CAP scores. On the same vein, it is unclear whether DTI measures were included in hierarchical stepwise regression. I wonder if the stepwise models may have picked up FA/MD instead of SANDI measures if they are given a chance. Overall, I hope the authors can discuss their findings also in this light of cost vs. benefit of adopting SANDI in future studies, which is an important topic for clinical trials.

(2) Similar to the above point, it is very important to consider how strong the biomarking signal is from SANDI measures compared to the good old striatal volume. Some plots seem to indicate that volumes still have the highest correlation with QMotor, and highest effect size in group comparisons. It would be helpful for the community to know where do the new SANDI measures stand compared to the most typically used volumes in terms of effect size.

(3) The diffusion measures are inevitably correlated to some degree. Please provide a correlation matrix in supplementary material including all DWI measures to enable readers to understand better how similar SANDI measures are between each other or vs. other DTI measures. Perhaps adding volumes to this correlation matrix may also be a good future reference.

(4) ISS stages:

(a) The online ISS calculator requires cut-offs derived from the longitudinal Freesurfer pipeline, while the authors do not have longitudinal data. Thus, the ISS classification might be inaccurate to some degree if the authors used the FS cross-sectional pipeline. Please review this issue and see if updated cut-offs should be used to classify participants.

(b) Were there really no participants with ISS 0 among 56 HD individuals, please clarify in the manuscript?

(c) A note on terminology that might be confusing to some readers. According to the creators of ISS, the ISS stages are created for research only, they are not used or applied in the clinic. On the other hand, the terms "premanifest" and "manifest" have a clinical meaning, typically based on the diagnostic confidence level. The assignment of ISS0-1 to premanifest and ISS2-3 to manifest may create some non-trivial confusion, if not opposition, in some segments the HD community. The authors can keep their current terminology but will need to at least clarify to the reader that this assignment is speculative, does not fully match the clinically-based categories, and should not be confused with similarly named groups in the previous literature.

Comments on revised version.

The authors have moved to address many points from reviewers. The manuscript had indeed become more objective, transparent, and to the point. The amount of information and analyses is large, which perhaps is inevitable when new methods are being tested for the first time in a neurodegenerative disease.

# Author comment: Author response

**DOI:** 10.7554/eLife.107661.3.sa3

**Authors:** Vasileios Ioakeimidis, Marco Palombo, Chiara Casella, Lucy Layland, Carolyn McNabb, Robin Schubert, Philip Pallmann, Monica Busse, Cheney Drew, Sundus Alusi, Timothy Harrower, Jane Davies, Anne Rosser, Claudia Metzler-Baddeley

**Affiliations:** Danish Research Centre for Magnetic Resonance Copenhagen Denmark; Cardiff University Cardiff United Kingdom; King's College London London United Kingdom; Cardiff University Cardiff United Kingdom; Cardiff University Cardiff United Kingdom; Georg Huntington Institut Muenster Germany; Cardiff University Cardiff United Kingdom; Cardiff University Cardiff United Kingdom; Cardiff University Cardiff United Kingdom; The Walton Centre for Neurology and Neurosurgery Liverpool United Kingdom; Royal Devon and Exeter NHS Trust Exeter United Kingdom; Cardiff University Cardiff United Kingdom; Cardiff University Cardiff United Kingdom; Cardiff University Cardiff United Kingdom

The following is the authors’ response to the original reviews.

**Reviewer #1:**

(1) The biological and/or mathematical meaning of the Soma and Neurite Density Imaging (SANDI) indices (apparent soma density, apparent soma size, extracellular water signal fraction, extracellular diffusivity, apparent neurite density, fractional anisotropy, and mean diffusivity) should be briefly introduced for those less familiar with this novel technique.

Further explanations about the biological and mathematical meaning of the SANDI indices were added to the introduction on page 6.

(2) The study implements a novel biophysical diffusion model that extends up-to-date methodologies and presents a significant potential for quantifying neurodegenerative processes of the grey matter of the human brain in vivo. The authors comment on the usefulness of this technique in other pathologies, but they exemplify it only with multiple sclerosis. Further development of this, building evidence, should be provided.

Clinical applications of SANDI have primarily focused on MS. However, since preparation of the manuscript, one study has been published reporting reductions in apparent soma density and white and grey matter specific differences in apparent soma size in amyotrophic lateral sclerosis (ALS) (Zeng et al., Eur J Radiol 2025, 10.1016/j.ejrad.2025.111981). These findings accord with the loss of motor neurons and glial responses in ALS. We have added this study to the introduction of SANDI on page 7.

(3) Why are the basal ganglia compared against thalami? The rationale of this decision is missing.

The thalami were selected as control regions based on the established trajectory of neurodegeneration in HD, which begins with early loss of medium spiny neurons in the striatum and later extends to surrounding structures, including the putamen and thalamus. Given that most participants in our study were at early disease stages, we assumed the thalami would remain relatively unaffected in this sample. This explanation has been added to the introduction on page 7.

(4) The use of bullet points is unusual for a scientific paper format.

Bullet points have been removed throughout the manuscript.

(5) The authors mention that they eroded the boundaries of the subcortical masks. Providing the details and parameters of this erosion would be beneficial.

Details of the default parameters of the FSL erode function that was used have been added to the method section on page 13.

(6) In the conclusion, the authors state that their results will bridge the gap between histopathological findings and in vivo imaging, but it would be helpful if they could briefly explain how they imagine such a bridge (e.g., which kind of comparisons or correlations) and whether there exists any literature in this regard so far.

We have added the following brief explanation to the conclusion on page 26: “Although conventional MRI lacks the resolution to directly capture histopathology, advanced biophysical models such as SANDI may help bridge this gap by providing biologically interpretable parameters that reflect tissue composition and capture histopathological changes in vivo.”

(7) The scale is missing in Figure 3.

The scale has been added to Figure 3.

(8) In general, the work would benefit from a better organization and potentially a smaller number of figures and tables.

The manuscript has been re-edited to improve the readability and organization throughout and the number of figures and tables were reduced by moving some of them to the Supplementary Material (old Tables 2 and 5 are now Supplementary Tables 2 and 3, old Figure 3 is now Supplementary Figure 1).

**Reviewer #2:**

Certain aspects of the study would benefit from clarification:

(1) Scanner and acquisition consistency: While HD data are from the WAND study, it is not clear whether controls were scanned on the same scanner or protocol. Given the use of model-derived metrics (especially SANDI), differences in scanner or acquisition could introduce confounds. From the text, the HD participants are explicitly said to come from the WAND study (a longitudinal HD cohort). On the other hand, while the HC participants are described as age-matched controls, the paper does not clearly state whether they were scanned in the same study (i.e., WAND), on the same scanner, or with the same acquisition protocol. This ambiguity is potentially problematic, especially since they use model-derived diffusion metrics that can be very sensitive to scanner hardware, gradient strengths, and protocol settings. If the WAND HD data were acquired on a specific scanner (e.g., 3T Connectom) and the HCs were not, then differences in SANDI/DTI metrics might reflect scanner bias, not disease pathology. This is particularly critical in SANDI, which is sensitive to high b-values and SNR. It would strengthen the manuscript to explicitly state whether the HD and control data were acquired using the same scanner model, sequence, and protocol, and ideally at the same site. If this were not the case, the authors should include this as a limitation and discuss any harmonization strategies applied (e.g., ComBat, covariate modeling, etc).

For harmonization and comparison purposes, HD and control data were acquired using the same strong gradient (300mT/m) 3T Connectom MRI system at CUBRIC with the same acquisition protocols and sequences. It should also be noted that the Connectom scanner has not had any software upgrades that could introduce scanner biases in data acquired at different time points. This is now made explicit on page 8 by stating that all MRI data for all participants were acquired on the same MRI system using the same acquisition protocols, and on page 10 by stating that all HD and HC MRI data included in our analyses were acquired on the same 3T Siemens Connectom scanner at CUBRIC using the same acquisition protocols described in this section.

Also, although it offers novel and biologically informative markers, widespread clinical translation still faces hurdles. For instance, the study used a 3T Connectom scanner (300mT/m gradients), which is not widely available. Reproduction of these results in standard 3T clinical scanners would be a great addition, in scenarios with lower resolution, less precise parameter recovery, and longer scans if SNR needs to be maintained.

We agree that for clinical adoption it is important to demonstrate that HD-related SANDI differences can also be detected on clinical MRI systems and do not require ultra-strong gradient imaging. While we have not collected such data in people with HD, we have demonstrated the feasibility of modelling SANDI metrics from multi-shell diffusion-weighted imaging acquired on a clinical 3T MRI (maximum b-value of 6,000 s/mm^2^) in healthy adults and people with MS (Schiavi et al 2023, https://doi.org/10.1002/hbm.26416). Furthermore, Zeng et al 2025, reported significant differences in SANDI metrics acquired on a 3T MRI Prisma system between individuals with ALS and healthy controls (maximum b-value of 3,000 s/mm^2^).

Two additional studies demonstrated that SANDI could be implemented and microstructural differences could be detected in MS using 3T scanners with standard gradient strength (Barakovic et al., 2024; Margoni et al., 2023). Collectively, these findings indicate that SANDI can be applied on clinical scanners, particularly as clinical systems move toward stronger gradient capabilities such as Siemens Magnetom Cima.X. These explanations can be found under the clinical implication section in the Discussion on page 25.

(2) Limitations of HD-ISS staging resolution and group separation:

The use of HD-ISS staging to anchor progression analyses is conceptually appropriate, but, in practice, the sample is quite limited.

(a) Only 26-27 out of 56 gene-positive participants could be assigned HD-ISS stages, and none were classified into stages 0 or 4. This restricts the interpretation of progression to a narrow clinical window (mostly stages 1-3) and excludes over 50% of the cohort.

(b) Furthermore, visual inspection of the scatter plots (e.g., Figures 3 and 4) reveals substantial overlap between stages 1 and 2, particularly in CAP100 and Q-Motor measures. This suggests that the separation between early disease stages may not be robust in this dataset, potentially due to limited power or phenotypic variability.

(c) The above may lead to claims based on progression across HD-ISS stages to be overinterpreted or underpowered

Despite this, the paper treats the staging as a reliable stratification for group comparisons. To improve clarity and transparency, I would recommend that the authors:

(a) Acknowledge that over 50% of the HD cohort could not be classified.

(b) Discuss whether those excluded differed from those included in key metrics.

(c) Explicitly comment on the substantial overlap between stages 1 and 2, and limit claims about progression unless such separation is statistically supported.

(d) Avoid overinterpreting staging-related effects without statistical support for group separability

Re (a-d) We have added to the study limitations on pages 23 ff that only 54% (30 out of 56) HD participants could be HD-ISS classified due to missing data, and provide an overview of demographic and clinical information for HD-ISS stages and unclassified individuals in Supplementary Table 1. We acknowledge that the combined groups (HD-ISS 0-1 versus HD-ISS 23) for exploratory group analyses did not represent discrete disease stages and that there was some overlap in imaging and behavioural features between them as illustrated in Figures 3, 4, and 7. We state explicitly that these exploratory findings should be interpreted with caution and require replication in larger, prospective cohorts before SANDI metrics can be considered as potential markers of disease progression.

(3) Clarify regression strategy and interpretational limits of SANDI-derived regressors: While the hierarchical regression strategy is broadly appropriate, several aspects would benefit from clarification to improve both interpretability and robustness of the findings. For example:

(a) Why were only a subset of SANDI parameters (fis and De) considered in the HC models (Figure 6), while additional metrics (fec and rs) were tested in HD models (Figures 7-8)? Including the same variables across groups could aid comparability?

The same SANDI indices were included in regression models for HD and HC groups, Figure 7-8 report only significant predictors. This has been clarified in the figure legend and on pages 14 of the manuscript.

(b) Were any checks for multicollinearity (e.g., variance inflation factors) conducted? Given known interdependencies among some SANDI parameters, I wonder whether some of the reported regression coefficients may be unstable or difficult to interpret.

Cross-correlation matrices between all imaging metrics for HD, HC, and total samples have been included to Supplementary materials Figure 3.

To improve transparency and interpretability, I suggest actions such as:

(a) SANDI metrics included in the models differ between HC and HD groups, reducing comparability. Consider using consistent full models across ROIs for comparison purposes, even if some predictors are not significant.

(b) Report the correlation structure between SANDI metrics within each group to assess multicollinearity (The potential impact of multicollinearity (e.g., between fis and rs) is not discussed)

(c) Explicitly acknowledge the limitations imposed by parameter degeneracy in the SANDI model and clarify how the authors ensured the biological interpretability of regression outputs in this context - Beta coefficients could reflect model instability or parameter degeneracy rather than true biological effects.

(a) The same SANDI metrics and age were included in the first regression models for HD and HC data. The first models only differed by the inclusion of TFC as estimate of disease burden for the HD data. HD and HC participants were not included in a single regression model, as our aim was not to perform formal between-group inference on regression coefficients. Instead, models were fitted separately to explore within-group associations and to descriptively compare patterns of relationships across groups. This approach avoids imposing identical model structures across groups that may differ in variance structure, disease burden, and biological coupling between SANDI metrics. We have clarified these points on page 13/14.

(b) We agree that multicollinearity is an important consideration when interpreting regression coefficients derived from microstructural models. To address this, we examined pairwise Spearman correlations between all imaging (SANDI, DTI, volume) metrics (averaged across ROIs), shown in the revised Supplementary Figure 2. As can be seen in the healthy control data, SANDI indices of apparent soma and neurite fractions showed a strong inverse correlation (rho = -0.92) and did not correlate with soma radius (rho = 0.1). All SANDI indices correlated only weakly with FA and volume and moderately with MD. This correlation pattern suggests that apparent soma density and radius capture distinct information about grey matter microstructure that differs from neurite fraction and is not captured by FA or volume. We note in HD participants a negative correlation between soma radius and fraction, and stronger correlations between SANDI metrics and volume measures. We would argue that these reflect disease-related reorganization of micro- and macro-structural relationships rather than uniform collinearity across groups. This information has been added to the Methods, Results and Discussion sections on pages 13, 19, and 21, 23ff.

(c) We agree that regression coefficients derived from interdependent microstructural parameters should be interpreted with caution, as they may reflect shared variance or partial parameter degeneracy rather than fully independent biological effects. For this reason, we do not interpret individual beta coefficients in isolation. Instead, our conclusions focus on the consistency and directionality of associations across regions and metrics, and on the overall feasibility and sensitivity of SANDI to detect biologically meaningful variation in HD. The observed correlation structure (Supplementary Figure 2) provides important context for these interpretations and supports a multivariate, pattern-based rather than univariate reading of the results. These points have been added to the Discussion on pages 23 ff. Please also refer to our response to point (5) below.

(4) Preprocessing order:

Gibbs ringing correction was applied after TOPUP and EDDY, which deviates from the commonly recommended order in diffusion MRI preprocessing. Since Gibbs artifacts are introduced by kspace truncation and affect the spatial domain, it is typically advised to perform Gibbs correction prior to geometric corrections like TOPUP and EDDY. This avoids potential blurring or propagation of ringing artifacts during resampling. Could the authors clarify the rationale for this ordering, and whether an early application of Gibbs correction was tested?

We agree that the application of Gibbs ringing correction prior to TOPUP and EDDY correction deviates from the commonly recommended order in diffusion MRI preprocessing. However, as some of the data included in this paper were preprocessed before this consensus was agreed in the literature, we kept the preprocessing order consistent for all datasets for harmonization and comparison purposes. We have since changed the order for subsequent preprocessing of the HDDRUM data and have found comparable FA maps for data processed with Gibbs ringing correction before and after TOPUP and EDDY correction.

(5) Expand on SANDI model assumptions:

SANDI is presented as being used for the very first time in this problem. However, a vague explanation is given: "using all the default settings". Given the novelty of applying SANDI in a clinical HD context, the manuscript would benefit from a discussion of the model's key assumptions and limitations. For instance:

(a) The potential degeneracy between fis and rs in the absence of protocol features (e.g., long Δ or high b) that can disambiguate them.

(b) Whether a dot compartment was included, and the implications of excluding it for the interpretation of rs or fis.

(c) The lack of exchange modeling or fixed stick diffusivity, and how these may bias compartment estimates (particularly in diseased or aging tissue).

(d) Any steps taken to verify robustness or identifiability (e.g., simulations, synthetic fitting). These issues are not flaws in the method, but they do affect how confident we can be in interpreting fis/rs as markers of neuron loss or glial hypertrophy, especially given the subtle group differences and the potential for biological heterogeneity in HD. Even a brief acknowledgment would strengthen the manuscript and provide useful context to readers less familiar with multicompartment modeling.

We thank the reviewer for this constructive suggestion and fully agree that, because this is the first application of SANDI in our clinical HD cohort, the manuscript should more explicitly describe the model assumptions, potential identifiability limitations under our protocol, and the implications for biological interpretation.

We have revised the Methods (pages 11-12) and Discussion (page 24) to (i) specify the exact SANDI implementation used (the SANDI MATLAB toolbox, available here), (ii) describe which components are included in the default formulation and the key modelling assumptions, and (iii) add a dedicated “Limitations and interpretability” paragraph addressing points (a–d) below. We also avoid the previous shorthand “default settings” and provide a clear description of the fitting setup.

“The SANDI model [Palombo M. et al, NeuroImage 2020] assumes three compartments, namely intra-neurite signal modelled as diffusion inside impermeable randomly oriented sticks, intra-soma signal modelled as restricted diffusion inside spheres, and extra-cellular signal modelled as Gaussian isotropic diffusion. The direction-averaged (or spherical mean) normalized diffusion signal has thus the following expression:

*S*(*b*) = *f_is_A_sphere_* (*b*, *r_s_*, *D_is_*) + *f_in_A_stick_* (*b*, *D_in_*) + *f_ec_A _ball_* (*b*, *D_e_*)

where f_in_ + f_is_+ f_ec_ = 1; A_stick_ and A_sphere_ are the normalized, directionally-averaged (or spherical mean) signals for restricted diffusion within neurites and soma, respectively and A_ball_ is the normalized, directionally-averaged (or spherical mean) signal of the extra-cellular space. The specific expressions are given in [Palombo M. et al. NeuroImage 2020]. The parameters estimated from the direction-averaged (or spherical mean) data are D_in_, proxy of the intra-neurite effective axial diffusivity; D_e_, proxy of the extracellular effective mean diffusivity; r_s μm. Rician noise with a distribution of standard deviations randomly sampled from the voxels within the brain mask of the noise map obtained using MPPCA denoising was added to account for realistic SNR levels and rectified noise floor. The loss function of the training was the mean squared error between predicted parameters and ground truth values. Model fitting provided maps of fin, fis, fe, Din, De and rs.”_

(a) Potential degeneracy between *f*_is_and *r*_s_. We agree that partial coupling (or degeneracy) between the soma fraction *f*_is_ and soma radius *r*_s_ is possible when the acquisition does not provide strong sensitivity to restricted sphere size (e.g., in the low b-values regime). Our protocol benefits from high b-values (up to 6000 s/mm^2^) enabled by the Connectom gradient system, which increases sensitivity to signal attenuation from restricted compartments and reduce the *f*_is_-*r*_s_ coupling/degeneracy. However, we acknowledge that the specific choice of fixed diffusion timing (in our case δ=7 ms, Δ=24 ms) can further modulate the *f*_is_-*r*_s_ coupling/degeneracy in a protocol-dependent way. To reflect this appropriately, we now explicitly state that *r*_s_ should be interpreted as an “apparent soma radius” under our protocol, and that our inferences focus on relative group differences and spatial patterns rather than absolute histological soma radii.

We have now added a paragraph in the limitations section acknowledging this point.

(b) Dot compartment. We did not include an explicit “dot” (immobile) compartment, because there is no evidence that in human in vivo this is required (see for example very low and negligible contribution provided in Tax C. et al. NeuroImage 2020). Accordingly, our fits did not include a dot term, and we now state this explicitly in the Methods. However, we would like to clarify that our fitting method (described in detail here) includes accurately the impact of Rician noise and thus it account for the corresponding rectified noise-floor that very often, in high b-values applications, is mistakenly associated with a “dot” compartment. Therefore, there is no expected bias on the estimated f_is_ and r_s_ due to not including a “dot” compartment.

(c) Exchange modelling and fixed stick diffusivity. We agree that SANDI, as implemented here, does not explicitly model inter-compartment exchange during the diffusion encoding and uses simplified representations of neurites (sticks), but the intra-stick diffusivity, D_in_, was not fixed but rather fitted. In diseased or aging tissue, deviations from these assumptions (e.g., altered membrane permeability) may bias compartment estimates. This has been investigated in dept in Schiavi S. et al. HBM 2023 (here), so we refer the redear to that. We have added an explicit limitation statement noting that HD-related microstructural changes (e.g., changes to membrane permeability) could affect model parameter fidelity, and thus *f*_is_and *r*_s_ should be treated as MRI-derived effective indices rather than direct quantitative measures of neuron loss or glial hypertrophy. Importantly, our analysis compares groups under an identical acquisition and fitting pipeline, so grouplevel contrasts remain informative even if absolute parameter values are biased.

(d) Robustness / identifiability checks. We agree that reporting robustness strengthens confidence, particularly given subtle effects and biological heterogeneity. The SANDI Matlab Toolbox we used extensively investigates model parameters robustness and identifiability using numerical simulations and synthetic signals accounting for the specific experimental protocol and noise distribution. An example of the results supporting the robustness / identifiability is reported in the Author response images. These results show that accuracy and precision of all SANDI model parameters, except D_in_, is very high (>~80%, Author response image 1)

**Author response image 1.** Analysis of the accuracy and precision of SANDI model parameters estimation. We simulated 10^4^ synthetic diffusion signals using the SANDI model with random combinations of five parameters: *f_neurite_*(*f_in_*), *f_soma_*(*f_is_*), *D_in_*, *R_soma_*(*r_s_*), and *D_e_*. Parameters were sampled uniformly from: *f_neurite_*, *f_soma_* ∈ [0,1]; *D_in_*, *D_e_* ∈[0.5,3.0] µm^2^/ms; *𝑅_soma_* 𝛜[1,12] µm. Rician noise with experimentally estimated variance was added, and the SANDI model was then fit to the noisy signals. For each parameter, we report the relative percentage error between estimated and ground-truth values as a function of the parameter value (normalized to [0,1]), together with goodness-of-fit (*R*^2^).

and sensitivity to changes as small as 5% in each of the model parameters is correctly captured (Author response image 2A), with small to negligible degeneracy (except, once again, for D_in_), even in presence of exchange (Author response image 2B).

**Author response image 2.** Sensitivity to 5% parameter modulations. The matrices show how a controlled perturbation in one parameter propagates into the estimated values of all model parameters. Each row corresponds to a 5% increase in the parameter on the y-axis; the resulting percentage change observed in each estimated parameter is reported along the x-axis. An ideal estimator would yield a purely diagonal matrix, with 5% on the diagonal and 0% elsewhere (no cross-talk). In (A), we used the same synthetic SANDI signals as in Figure 1. In (B), we additionally generated 10^4^ synthetic signals incorporating neurite–extra-cellular exchange using the NEXI model [https://doi.org/10.1016/j.neuroimage.2022.119277] and an exchange time representative of human cortex (𝜏_ex_ ≈ 30 ms) [https://doi.org/10.1162/imag_a_00104].

We have therefore revised the manuscript language to be more precise and appropriately cautious, describing *f_is_* and *r_s_* as apparent compartment indices and explicitly discussing potential confounds (e.g., parameter coupling, and unmodelled exchange), while clarifying the value of SANDI for detecting reproducible group-level microstructural differences in HD.

(6) Clarify "not-classified" group in figures:

It is not clear to me what the "not-classified" groups shown in Figures 3-4 represent, what criteria determined their inclusion, and whether their inclusion affects the comparability or interpretability of staging-based analyses

We have added to the legends of Figures 3 and 4 that not-classified refers to HD participants who could not be HD-ISS classified due to missing clinical data or their CAG repeat falling within the 36-40 range. As correlation analyses were conducted across the whole HD sample though, these datapoints were included in the scatterplot.

(7) Figure labeling:

There appears to be a mismatch between figure numbering and captions around Figures 3-4. Please ensure alignment.

Mismatch between figure numbering and captions has been corrected.

Minor suggestions:

(1) Figures 1-2:

(a) Label axis values meaningfully, e.g., negative vs. positive instead of 0 vs 1.

(b) Add units to MD axes (e.g., ×10⁻⁴ mm²/s).

(c) Figure 6 colors: Consider improving the color distinction between "Age" and "fis" predictors, which are currently hard to differentiate.

The suggested adjustments have been made to Figures 1, 2, 5 and 6 and Figure 2 legend.

(c) Discuss why apparent soma size decreases in some ROIs (e.g., pallidum), if unexpected.

We offer the following speculation about the reduced soma size in the pallidum (pages 20/21): Changes in apparent soma size may reflect alterations in neural and glial cell proportions and/or morphology, including astrocyte and microglia swelling in response to neurodegeneration and soma shrinkage preceding neuronal cell death. Thus, increased apparent soma size in the striatum may indicate HD-related reorganisation of cell types driven by MSN loss and reactive glial cell swelling, whereas smaller soma size in the pallidum may result from infiltration of smaller glia cells prior to secondary neuronal loss following striatal MSN degeneration.

**Reviewer #3:**

(1) An important question is whether the SANDI measures, which require an expensive scanner and elaborate processing, are better biomarkers than the more traditional DTI measures. Can the authors compare the effect size of FA/MD with SANDI measures? In some of the plots and tables, FA/MD seem to have comparable, if not higher, correlations with QMotor or CAP scores. On the same vein, it is unclear whether DTI measures were included in hierarchical stepwise regression. I wonder if the stepwise models may have picked up FA/MD instead of SANDI measures if they are given a chance. Overall, I hope the authors can discuss their findings also in this light of cost vs. benefit of adopting SANDI in future studies, which is an important topic for clinical trials.

Effect sizes (ES) of group differences in all microstructural indices can be found in Table 4. ES of DTI and SANDI indices in the caudate and putamen were broadly comparable with a trend for MD showing larger ES (FA: r_rb_ = 0.38 -0.55, MD: r_rb_ = 0.51 -0.61, f_is_: r_rb_ = 0.32 -0.45, r_s_: r_rb_ = 0.45 0.53).

This information is now reported in the result section on pages 15/16 and is being discussed in light of cost versus benefit considerations on pages 21 and 25.

(2) Similar to the above point, it is very important to consider how strong the biomarking signal is from SANDI measures compared to the good old striatal volume. Some plots seem to indicate that volumes still have the highest correlation with QMotor and the highest effect size in group comparisons. It would be helpful for the community to know where the new SANDI measures stand compared to the most typically used volumes in terms of effect size.

Effect sizes (ES) of group differences in volumes can be found in Table 2. ES in caudate and putamen volumes ranged between r_rb_ = 0.49 -0.55 and were comparable to the ES of apparent soma size r_rb_ = 0.45 -0.53 but slightly larger than ES of soma density r_rb_ = 0.32 -0.45.

This information is now reported in the result section on page 15/16 and is being discussed on pages 21 and 25.

(3) The diffusion measures are inevitably correlated to some degree. Please provide a correlation matrix in the supplementary material, including all DWI measures, to enable readers to better understand how similar SANDI measures are to each other or vs. other DTI measures. Perhaps adding volumes to this correlation matrix may also be a good future reference.

We have added cross-correlation matrices between all imaging measures (SANDI, DTI, Volumes) for the total sample as well as for HC and HD participants separately to the Supplementary material (Figure 3), providing an overview of the shared variance within SANDI parameters and between SANDI and DTI and volume metrics for each group.

(4) ISS stages:

(a) The online ISS calculator requires cut-offs derived from the longitudinal Freesurfer pipeline, while the authors do not have longitudinal data. Thus, the ISS classification might be inaccurate to some degree if the authors used the FS cross-sectional pipeline. Please review this issue and see if updated cut-offs should be used to classify participants.

We acknowledge that our HD-ISS classifications may have been biased due to the use of crosssectional rather than longitudinal FreeSurfer v6 volumes (page 23).

(b) Were there really no participants with ISS 0 among the 56 HD individuals? Please clarify in the manuscript.

We classified four individuals as ISS 0 based on their caudate and/or putamen z-scored volumes falling below 2SD of the healthy control mean. These analyses are described on pages 14-15 and were based on the cross-sectional data of this study.

(5) A note on terminology that might be confusing to some readers. According to the creators of ISS, the ISS stages are created for research only; they are not used or applied in the clinic. On the other hand, the terms "premanifest" and "manifest" have a clinical meaning, typically based on the diagnostic confidence level. The assignment of ISS0-1 to premanifest and ISS2-3 to manifest may create some non-trivial confusion, if not opposition, in some segments of the HD community. The authors can keep their current terminology, but will need to at least clarify to the reader that this assignment is speculative, does not fully match the clinically-based categories, and should not be confused with similarly named groups in the previous literature.

To avoid confusion about terminology, we have removed the labels “premanifest” versus “manifest” throughout the manuscript. We refer to HD-ISS 0-1 and HD-ISS 2-3 when referring to the exploratory comparisons between HD-ISS stages.

(6) The population in the study seems to be obtained from different other studies or research projects, and there are missing scores for several participants due to the retrospective nature of sample gathering for the analyses. Please state clearly that this study was done with retrospective data to properly justify why there are missing data. Also, and this is important, please clarify for the reader whether there was any temporal bias in the acquisition of data of a certain group (HD) vs. another (HC). It is important to rule out that there were no scanner changes or upgrades that may confound the reported group differences.

We can confirm there were no Connectom scanner changes or upgrades that may have confounded the reported group differences. This was added to the image acquisition section on page 10. We have added to the participant section on page 9 that data were retrospectively pooled from separate studies and explain this was the reason why HD-ISS classification was only available for a subset of participants.

(7) Several of the significant results with SANDI scores seem to be driven by a subgroup of HD individuals that are more clearly different than the healthy control distribution. Not sure if this may help, but one idea the authors can consider is to check if HD individuals that deviate more than 2 SDs from the healthy control distribution of SANDI scores have also worse QMotor, worse atrophy, or higher CAP scores than those HD individuals that are practically within the 2SD boundary distribution of HDs. This is another way of showing that the new measures have potential for application in individualized medicine (the MRI Z score of a patient as a proxy of the clinical deterioration). It is not a request to authors but just a suggestion for their consideration.

The data points in the scatterplots of Figures 3, 4, and 7 have now been color-coded according to HD-ISS stage, showing a stage-related worsening of microstructural and volumetric imaging markers and Q-Motor performance.

(8) The variance explained in hierarchical regression is obtained by fitting models within the sample, and can be subject to overfitting. In the absence of a more robust cross-validated R2, the authors may want to at least briefly inform the reader that the current approach can be subject to overfitting and does not represent a true out-of-sample R2.

We have added this point to the study limitations in the Discussion section on page 23.

(9) There are two Figure 3 labels, and all figures thereafter do not match the manuscript.

The Figure numbering has been corrected.

(10) In (the currently labelled) Figure 8, there seem to be fewer than 56 data points in the scatterplots. Is there a reason why not all 56 HD individuals do not have the CAP100 score available? CAP needs only CAG and age, which all HD gene carriers should have, to be included in the study.

Inclusion criteria for individuals with HD for the HD-DRUM project were a positive genetic test for the presence of the mutant huntingtin allele (CAG length ≥ 36 repeats) and/or a clinical diagnosis of HD. Thus, for a small number of participants CAG was not available for the calculation of CAP100 score.
